# Phenotypic characterization of circulating tumor cells in the peripheral blood of patients with small cell lung cancer

**DOI:** 10.1371/journal.pone.0181211

**Published:** 2017-07-18

**Authors:** Ippokratis Messaritakis, Eleni Politaki, Athanasios Kotsakis, Eleftheria-Kleio Dermitzaki, Filippos Koinis, Eleni Lagoudaki, Anastasios Koutsopoulos, Galatea Kallergi, John Souglakos, Vassilis Georgoulias

**Affiliations:** 1 Laboratory of Tumor Cell Biology, Medical School, University of Crete, Heraklion, Greece; 2 Department of Medical Oncology, University General Hospital of Heraklion, Crete, Greece; 3 Department of Pathology, University General Hospital of Heraklion, Crete, Greece; University of South Alabama Mitchell Cancer Institute, UNITED STATES

## Abstract

**Background:**

To evaluate the phenotypic heterogeneity of circulating tumor cells (CTCs) based on the expression of proliferative, apoptotic and Epithelial-to-Mesenchymal Transmission (EMT) markers during front-line treatment in patients with small cell lung cancer (SCLC) and to evaluate their clinical relevance.

**Methods:**

CTCs from 108 chemotherapy-naïve patients with SCLC were analyzed by double immunofluorescence staining using anti-Ki67, anti-M30, anti-Vimentin along with anti-CKs antibodies. In 83 patients CTCs were also enumerated using the CellSearch.

**Results:**

Sequential samples were available from 76 and 48 patients after one-treatment cycle and on disease progression (PD), respectively, for immunofluorescence and from 50 and 36 patients after one-cycle and on PD, respectively, for CellSearch. At baseline, 60.2% of the patients had detectable CTCs by either method. Both proliferative (CK67^+^) and non-proliferative (Ki67^-^), apoptotic (M30^+^) and non-apoptotic (M30^-^) as well as EMT (Vim^+^) CTCs were present in the same patient. Among 22 patients without detectable CTCs by CellSearch, CK^+^/Ki67^+^ and CK^+^/Vim^+^ CTCs could be detected in 6 (27.3%) and 6 (27.3%) patients, respectively. One-chemotherapy cycle reduced both the incidence of detection (*p<*0.001) and the absolute number (*p<*0.001) of CTCs; conversely, on PD both the incidence of detection and the number of CTCs were significantly increased (*p =* 0.002 and *p* = 0.04, respectively). Multivariate analysis revealed that the increased number of Vim^+^ CTCs at baseline and of non-apoptotic CTCs on PD could be emerged as independent prognostic factors associated with decreased OS(*p =* 0.009 and *p =* 0.023, respectively).

**Conclusions:**

CK^+^/Ki67^+^, CK^+^/M30^+^ and CK^+^/Vim^+^ CTCs represent distinct subpopulations of CTCs in patients with SCLC, can be detected even in the absence of detectable CTCs by CellSearch; CK^+^/Ki67^+^ and CK^+^/Vim^+^ CTCs are associated with unfavorable clinical outcome.

## Introduction

Small Cell Lung Cancer (SCLC) is an aggressive disease accounting for about 13% of all lung cancer cases [1,2]. Front-line chemotherapy for extensive stage disease and chemo-radiotherapy for limited disease represent the standard of care and are associated with a high response rate; however, the disease relapses [3] and only 20–30% and 1–3% of patients with limited and extensive disease, respectively, survive for at least 5 years [4,5].

The high metastatic potential of the disease is due to the dissemination of tumor cells through the hematogenous and/or the lymphatogenous vasculature. The presence of tumor cells in the peripheral blood (circulating tumor cells; CTCs) and bone marrow aspirates (disseminated tumor cells; DTCs) has already been described in cancer patients [6,7,8,9,10,11,12]. Moreover, several studies have reported the prognostic relevance of CTCs in various tumor types such as breast, colon, prostate, non-small cell lung cancer and SCLC [13,14,15,16,17]. In SCLC patients, the detection of CTCs before the initiation of treatment as well as post-treatment and at the time of clinical relapse has been shown to be associated with a worse overall survival [16,18,19,20,21]. In addition, Hou *et al*. [16], using the ISET technology, reported the presence of circulating tumor microemboli (CTM) which were also associated with unfavorable clinical outcome; in addition, they reported that up to 20% of the solitary CTCs but none of the CTCs comprising to CTMs were apoptotic [16].

The CellSearch platform, based on the immunomagnetically enrichment of CTCs expressing the epithelial cell adhesion molecule (EpCAM), is frequently used for the detection and enumeration of CTCs [14]. However, during tumor cell dissemination the cells undergo epithelial-to-mesenchymal transition (EMT) which is characterized by down modulation of their epithelial phenotype and the acquisition of a mesenchymal phenotype [22,23]. We have recently reported that CTCs from patients with early and metastatic breast cancer express putative stemness and EMT markers, such as vimentin, N-cadherin, fibronectin, alpha-smooth muscle actin [24,25,26], suggesting their EMT status. Therefore, it seems that the CellSearch assay fails to detect CTCs expressing an EMT phenotype. This could explain, at least partly, the high heterogeneity of CTCs which could be, probably, linked to their metastatic potential. Additionally, the characterization of patients’ CTCs viability might have prognostic and predictive value since the presence of exclusively apoptotic CTCs could be a favourable prognostic factor; in contrast, as has been shown in different tumor types, the prevalence of proliferating CTCs could be related to poor patient’s outcome [24,27,28,29,30,31,32,33].

The current study was designed in order to investigate the heterogeneity of CTCs in patients with SCLC before and during front-line treatment and to evaluate its association with the clinical outcome.

## Materials and methods

### Patient samples and cytospin preparation

Peripheral blood (20 ml in EDTA and 7.5 ml in CellSearch Save preservative tubes; Raritan, NJ, USA) was obtained from 108 consecutive chemotherapy-naïve patients with SCLC. All blood samples were obtained at the middle-of-vein puncture after the first 5 ml of blood were discarded to avoid contamination with epithelial cells from the skin. All patients enrolled in the study were diagnosed in the Department of Medical Oncology of the University Hospital of Heraklion, Crete, Greece and had histologically confirmed SCLC. Patients with extensive-stage disease (ED-SCLC) received up to 6 cycles of standard chemotherapy [etoposide and cisplatin; (VP16/CDDP)] while patients with limited-stage disease (LD-SCLC) were treated with concurrent VP16/CDDP and involved field radiotherapy and prophylactic cranial irradiation. The study has been approved prospectively, prior to the start of the study, by the Ethics and Scientific Committees of University Hospital of Heraklion, Crete, Greece, and all patients signed written informed consent to participate in the study.

Peripheral blood mononuclear cells (PBMCs) were isolated by Ficoll–Hypaque density gradient (*d* = 1,077 g/ml; Sigma-Aldrich Chemie GmbH, Germany) centrifugation. Aliquots of 5 × 10^5^ PBMCs were cyto-centrifuged at 2,000 rpm for 2 min on glass microscope slides [24,25], were air dried and stored at −80°C until use. Two slides (10 × 10^5^ PBMCs) from each patient were analyzed at each time point.

### Detection of CTCs using the CellSearch platform

For the enumeration of CTCs using the CellSearch, peripheral blood was maintained at ambient temperature and processed within 72 h. The standardized CellSearch technique (Veridex LCC, Raritan, NJ) for the detection of CTCs was performed according to the manufacturer’s instructions. CTC morphology was confirmed in all cases and analysis was performed with the CellTracks Analyser II by an experienced biologist (E.P.) and according to the manufacturer’s instructions. In brief, the CellSearch kit contains ferrofluid particles coated with anti-EpCAM antibodies, phycoerythrin conjugated anti-CK antibodies recognizing cytokeratins (8, 18 and/or 19) to specifically identify epithelial cells and allophycocyanin-conjugated anti-CD45 antibody in order to identify white blood cells. Nuclear dye (4',6-diamidino-2-phenylindole/DAPI) was also added so as to fluorescently label the cell nuclei. In the final processing step, the selected cells were transferred automatically to a cartridge in a MagNest cell presentation device after an incubation of at least 20 min in the dark and at room temperature. The MagNest was then moved to Cell Tracks Analyzer II, which contains a semi-automated fluorescent microscope (4 fluorescent filter cubes) which captures images of fluorescently labeled cells that are immunomagnetically selected and aligned, covering the entire surface of the cartridge. The images are presented in a gallery format to the operator which classifies according to predetermined criteria (specified by Veridex) for the presence of CTCs. A cell is classified as epithelial cell (CTC) if it meets the following: Nearly round to oval morphology, visible nucleus within the cytoplasm, cytokeratin-phycoerythrin positive, DAPI positive, CD45-allophycocyanin negative and size of at least 4 μm. Results are expressed as number of CTCs/7.5 ml blood.

### Double immunofluorescence assay

The presence of CTCs in PBMCs’ cytospin preparations was investigated using monoclonal antibodies against Ki67 (a proliferation marker; Abcam, Cambridge, UK), M30 (an apoptosis marker CytoDEATH fluorescein; Roche, Manheim, Germany) and Vimentin (an EMT marker; Santa Cruz, CA, USA). In addition, the epithelial origin of the cells was confirmed using the mouse A45-B/B3 antibody (detecting CK8, CK18 and CK19 and will be referred as CK in the text) (Micromet, Munich, Germany). The cytomorphological criteria proposed by Meng *et al*. (i.e. high nuclear/cytoplasmic ratio, larger cells than white blood cells) were used to characterize a CK-positive cell as a CTC [34]. Control experiments for the sensitivity and the specificity of the CK antibody have already been reported [35,36,37]. Briefly, PBMCs’ cytospins were fixed with ice cold aceton:methanol 9:1 (v/v) for 20 min. The incubation period for all primary and secondary antibodies was 1 h. Ki67 and Vimentin were labelled with the anti-rabbit Alexa 555 (Molecular Probes, Invitrogen, Carlsbad, USA), M30 was a fluorescein-conjugated mouse antibody and A45-B/B3 was detected using the corresponding secondary fluorescein isothiocyanate (FITC) fluorochrome or the anti-mouse Alexa 555 (Molecular Probes). Finally, DAPI antifade reagent (Molecular Probes) was, added to each sample for cell nuclear staining. The omission of the first antibodies (anti-Ki67, anti-M30, anti-Vim, anti-CK) has been always, used in control experiments. Slides were analyzed using a fluorescent microscope (Leica DM 2500, Heidelberg, Germany). Results are expressed as number of CTCs/10^6^ PBMCs.

### Cell lines

The human SKBR3, MDA-MB231 and HeLa cell lines were obtained from the American Type Culture Collection (ATCC, Manassas, VA, USA) and used as positive controls as follows: SKBR3 cells treated in the presence or absence of staurosporine were used as positive controls for CK and M30 expression, as it has been previously described [33,38]. Cyto-centrifuged MDA-MB231 cells were used as positive controls for CK and Ki67 expression. The HeLa adenocarcinoma cells were used as positive controls for CK and vimentin expression. In addition, all cell lines were double stained with anti-CD45 (Common Leukocyte Antigen; Santa Cruz) and either anti-Ki67 or anti-M30 or anti-Vim antibodies in order to exclude possible ectopic expression on such cells. To determine the sensitivity of the method, all cell lines were spiked in peripheral blood obtained from healthy individuals and the PBMCs, obtained after Ficoll-Hypaque density centrifugation, were used to prepare cytospins, as per patients’ samples.

SKBR3 cells were cultured in McCoy’s 5A GlutaMAX supplemented with 10% fetal bovine serum (FBS) (Gibco BRL Life Technologies, Rockville, USA). MDA-MB231 cells were cultured in Dulbecco’s modified Eagle’s medium (DMEM) GlutaMAX supplemented with 10% FBS. MDA-MB231 cells were cultured in Dulbecco’s modified Eagle’s medium (DMEM) GlutaMAX supplemented with 10% FBS. HeLa cells were cultured in 1:1 (vol/vol) DMEM (Gibco-BRL) supplemented with 10% foetal bovine serum (FBS) (Gibco-BRL), 2 mmol L-glutamine (Gibco-BRL) and 50 mg/mL penicillin/streptomycin (Gibco-BRL). All cells were maintained in a humidified atmosphere of 5% CO_2_ in air. Sub-cultivation of all cell lines was performed using 0.05% trypsin and 5 mmol ethylene-diamine-tetra-acetic acid (EDTA) (Gibco BRL). All experiments were performed during the logarithmic growth phase of each cell line. The detachment of the cells and the dispersion of the adherent cells into single cells prior cyto-centrifugation were performed again by enzymatic digestion using the 0.05% trypsin/EDTA solution (Gibco BRL). As soon as cells have detached, culture medium was added to the flask for the trypsin inactivation, due to the presence of FBS. Since, any detachment technique (thermal, mechanical or enzymatic) might influence the cell behaviour and antibody binding, the determination of the optimal antibody concentration was a necessary step.

### Study design and statistics

This is a prospective, single institution study, investigating the expression of the Ki67, M30 and Vim in CTCs from patients with SCLC before the initiation of front line treatment, after one-treatment cycle and on disease progression (PD). Because of the observational nature of the study, there was no specific sample size estimation. Progression-free survival (PFS) and overall survival (OS) were calculated from the day of diagnosis to the first clinical or radiologic evidence of PD and/or development of a new metastatic lesion and death, respectively. The detection of CTCs was done blindly to clinical data. The potential association between baseline clinico-pathological characteristics and the detection of CTCs was compared with the 2-sided Fisher exact test for categorical variables. Coefficient correlation between variables was performed using the Spearman test. The association of risk factors with time-to-event endpoints was analyzed with the log rank test and the Kaplan–Meier method was used to plot the corresponding PFS and OS curves. Univariate and multivariate Cox proportional hazards regression models with hazard ratios (HR) and 95% CIs were used to assess the association between potential prognostic factor and PFS or OS. Statistical significance was set at *p =* 0.05. All statistical analysis was performed using the SPSS v. 20 software.

## Results

### Ki67, M30 and Vimentin expression in tumor cell lines and blood donors’ PBMCs

Immunofluorescent staining revealed that 95%±5% MDA-MB231 were CK^+^/Ki67^+^, 95%±5% SKBR3 were CK^+^/M30^+^ and 95%±5% HeLa cells were CK^+^/Vim^+^ (mean+/-SD values from 5 experiments). The expression of all markers was subsequently investigated in PBMC cytospins from 10 healthy blood donors; Ki67, M30 and Vim were found to be expressed in PBMCs, however, there were no detectable CK^+^/Ki67^+^, CK^+^/M30^+^ or CK^+^/Vim^+^ double stained cells.

### Patients’ characteristics

From 11/2010 to 05/2015, CTCs from 108 consecutive patients with SCLC were characterized by immunofluorescence. The patients’ characteristics are listed in Table 1. Their median age was 66 years, 91 (84.3%) were males, 63 (58.3%) had a good PS (ECOG) 0–1, 71 (65.7%) had ED-SCLC, and 79 (73.1%) had increased lactate dehydrogenase (LDH) serum levels; moreover, 12 (11.1%) patients had brain, 40 (37.0%) liver and 32 (29.6%) bone metastases on diagnosis. Thirty eight (35.2%) patients received concurrent chemo-radiotherapy for the treatment of LD-SCLC. An objective response (CR and PR) was achieved in 77 (71.3%) patients. CTC enumeration before treatment, using the CellSearch assay, was performed in 83 (76.9%) patients. In the remaining 25 patients, the failure to enumerate CTCs at baseline was due to various technical reasons (S1 Fig). High CTC number (≥5 CTCs/7.5 ml) could be detected in 50 (60.2%) patients (Table 2 and S1 File) in agreement with previous studies [18,19].

**Table 1:** Clinical characteristics of SCLC patients.

	Immunofluorescence N (%) (n = 108)	CellSearch N (%) (n = 83)		*p-*value
		≥5 CTCs	<5 CTCs	
**Age (median)**	66 (range, 44–82)			
**Gender**				
**Male**	91 (84,3%)	42 (50,6%)	28 (33,7%)	0,586
**Female**	17 (15,7%)	8 (9,6%)	5 (6,0%)	
**PS**				
**0–1**	63 (58,3%)	23 (27,7%)	27 (32,5%)	0,001
**≥2**	45 (41,7%)	27 (32,5%)	6 (7,2%)	
**Stage**				
**Limited disease (LD)**	37 (34,3%)	8 (9,6%)	21 (25,3%)	<0,001
**Extensive disease (ED)**	71 (65,7%)	42 (50,6%)	12 (14,5%)	
**LDH**				
**High**	79 (73,1%)	43 (52,4%)	17 (20,7%)	0,001
**Low**	27 (25,0%)	8 (8,6%)	15 (18,3%)	
**Unknown**	2 (1,9%)	-	-	
**Liver Metastases**				
**Yes**	40 (37,0%)	28 (33,7%)	3 (3,6%)	<0,001
**No**	66 (61,1%)	21 (25,3%)	30 (36,1%)	
**Unknown**	2 (1,9%)	1 (1,2%)	0 (0,0%)	
**CNS at diagnosis**				
**Yes**	12 (11,1%)	6 (7,2%)	4 (4,8%)	0,787
**No**	92 (85,2%)	43 (51,8%)	29 (34,9%)	
**Unknown**	4 (3,7%)	1 (1,2%)	0 (0,0%)	
**Bone Metastases**				
**Yes**	32 (29,6%)	23 (27,7%)	1 (1,2%)	<0,001
**No**	71 (65,7%)	25 (30,1%)	30 (36,1%)	
**Unknown**	5 (4,6%)	2 (2,4%)	2 (2,4%)	
**Response**				
**CR/PR**	77 (71,3%)	32 (38,6%)	28 (33,7%)	0,034
**SD**	11 (10,2%)	6 (7,2%)	4 (4,8%)	
**PD**	14 (13,0%)	9 (10,8%)	1 (1,2%)	
**NE**	6 (5,6%)	3 (3,6%)	0 (0,0%)	

The patients’ demographics and clinical characteristics are presented in the table. All patients were evaluated for CTC detection according to IF. CellSearch analysis was performed in 83 patients and the results were correlated with patients’ clinical characteristics.

**Table 2:** Detection of different sub-populations of CTCs during treatment.

	Baseline		Post1		Progression	
	N of +ve patients (%)	Median (range)	N of +ve patients (%)	Median (range)	N of +ve patients (%)	Median (range)
**CellSearch**	50/83 (60,2%)	14 (0–10000)	16/55 (29,1%)*	0 (0–3459)†	29/44 (65,9%)^a^	43 (0–11143)‡
**CK**^**+**^**/Ki67**^**+**^	57/108 (52,8%)	2 (0–149)	31/76 (40,8%)**	0 (0–129)††	45/48 (93,8%)^b^	21 (0–246)‡‡
**CK**^**+**^**/Ki67**^**-**^	65/108 (60,2%)	2 (0–207)	23/76 (30,3%)***	0 (0–124)†††	33/48 (68,8%)^c^	7 (0–124)‡‡‡
**CK**^**+**^**/M30**^**+**^	18/108 (16,7%)	0 (0–14)	5/76 (6,6%)****	0 (0–9)	7/48 (14,6%)	0 (0–15)
**CK**^**+**^**/M30**^**-**^	63/108 (58,3%)	3 (0–267)	25/76(32,9%)*****	0 (0–139)††††	38/48 (79,2%)^d^	16 (0–199)‡‡‡‡
**CK**^**+**^**/Vim**^**+**^	57/108 (52,8%)	3 (0–209)	25/76 (32,9)******	0 (0–133)†††††	46/48 (95,8%)^e^	22 (0–207)‡‡‡‡‡
**CK**^**+**^**/Vim**^**-**^	61/108 (56,5%)	1 (0–221)	22/76 (28,9%)	0 (0–126)††††††	37/48 (77,1%)^f^	4 (0–126)‡‡‡‡‡‡

The detection of different sub-populations of CTCs at baseline, after one treatment cycle and on disease progression are presented here, as revealed by the CellSearch system and immunofluorescence double staining. The number and percentage of patients with detectable CTCs, as well as the median absolute number and range of detected CTCs are shown.

p-value: Baseline vs Post 1st cycle:

*<0,001;

**0,001;

***<0,001;

****0,008;

*****<0,001;

******<0,001;

^†^<0,001;

^††^<0,001;

^†††^<0,001;

^††††^<0,001;

^†††††^<0,001;

^††††††^<0,001

*p-*value: Post 1st vs Progression:

^a^0,002;

^b^0,008;

^c^<0,001;

^d^0,001;

^e^<0,001;

^f^<0,001;

^‡^0,04;

^‡‡^<0,001;

^‡‡‡^0,006;

^‡‡‡‡^<0,001;

^‡‡‡‡‡^<0,001;

^‡‡‡‡‡‡^<0,001

### Detection of CTCs subpopulations by immunofluorescence and CellSearch before the initiation of systemic treatment

Representative patients’ CTCs stained with anti-Ki67, anti-M30 and anti-Vim antibodies are presented in S2 Fig. Table 2 demonstrates that 57 (52.8%) and 65 (60.2%) patients had both proliferative (CK^+^/Ki67^+^) and non-proliferative (CK^+^/Ki67^-^) CTCs at baseline (S1 File). In addition, most patients (58.3%) had non-apoptotic (CK^+^/M30^-^) CTCs whereas CK^+^/Vim^+^ CTCs could be detected in 57 (52.8%) patients, suggesting that these cells undergo EMT (S1 File).

Among the 83 patients tested for CTCs using the CellSearch platform, 50 (60.2%) were considered as positive at baseline with ≥5 CTCs/7.5 ml (median 14 CTCs/7.5 ml; range, 0–10.000) (Table 2 and S1 File) and 33 (39.8%) were considered as negative (<5 CTCs/7.5 ml; range, 0–4). Among the 33 patients with a low CTC number, 11 had ≥1 CTC/7.5 ml (Table 1).The detection of CTCs was correlated with patients’ PS (*p* = 0.001), disease extent (*p*<0.001), concurrent chemo-radiotherapy in LD-SCLC (*p*<0.001), LDH levels (*p* = 0.001), liver or bone metastases (*p*<0.001) and treatment response (*p* = 0.034) (Table 1).

The phenotypic analysis of CTCs in the group of patients tested by CellSearch indicated that the detection of CTCs with a proliferative phenotype was independent of the detection of CTCs using the CellSearch whilst non-proliferative phenotype was mainly observed in patients with high CTC number (*p* = 0.088 and <0.001, respectively) (Table 3). In addition, both CK^+^/M30^+^ and CK^+^/M30^-^ CTCs were mainly observed in patients with ≥5 CTCs/7.5 ml (p = 0.002 and p<0.001, respectively) (Table 3). Finally, 56.5% of the patients had also CK^+^/Vim^-^ CTCs at baseline and this cell population was mainly observed in patients with a high CTCs’ number (*p*<0.001) (Tables 2 and 3).

**Table 3:** Heterogeneity of CTC sub-populations according to CellSearch positivity before treatment initiation.

	CellSearch platform (N = 83)		
Phenotype	≥5 CTCs (N = 50)	<5 CTCs (N = 33)	*p*-value
**CK**^**+**^**/Ki67**^**+**^	27 (32,5%)	12 (14,5%)	0,088
**CK**^**+**^**/Ki67**^**-**^	49 (59,0%)	2 (2,4%)	<0.001
**CK**^**+**^**/M30**^**+**^	11 (13,3%)	0 (0,0%)	0,002
**CK**^**+**^**/M30**^**-**^	42 (82,1%)	4 (4,8%)	<0,001
**CK**^**+**^**/Vim**^**+**^	26 (31,3%)	13 (15,7%)	0,184
**CK**^**+**^**/Vim**^**-**^	42 (50,6%)	4 (4,8%)	0,001

The detection of different CTC sub-populations at baseline according to the positivity of the CellSearch is shown and presents high heterogeneity.

### Detection of subpopulations of CTCs by immunofluorescence in patients with undetectable CTCs by CellSearch

Among the 22 patients with undetectable CTCs by the CellSearch (0 CTCs/7.5 ml), 6 (27.3%), 0 (0.0%) and 6 (27.3%) of them had CK^+^/Ki67^+^, CK^+^/M30^+^ and CK^+^/Vim^+^ CTCs, respectively (S1 Table and S1 File). Moreover, in 13 patients with detectable CTCs by immunofluorescence but not by CellSearch, all the subpopulations of CTCs were present at baseline (S2 Table and S1 File); it is to note that in these particular patients, cell staining with an anti-EpCAM antibody could not reveal the presence of CK^+^/EpCAM^+^ or Vim^+^/EpCAM^+^ CTCs.

### Effect of treatment on CTCs

The administration of one-treatment cycle resulted in a significant decrease of the patients with a high CTC number as well as of the absolute number of CTCs compared to the pre-treatment values (S3 Fig and Table 2; *p*<0.001). Moreover, one treatment cycle resulted to: (i) a significant decrease of the number of patients with proliferative (CK^+^/Ki67^+^) (40.8%; *p* = 0.001) and non-proliferative (CK^+^/Ki67^-^) (30.3%; *p*<0.001) CTCs compared to baseline; (ii) a significant decrease of patients with apoptotic (CK^+^/M30^+^) (6.6%; *p* = 0.008) and of non-apoptotic (CK^+^/M30^-^) CTCs (32.9%; *p*<0.001) compared to baseline; and (iii) a significant decrease of the number of patients with CK^+^/Vim^+^ CTCs (32.9%; *p*<0.001) but not of CK^+^/Vim^-^ CTCs compared to baseline (S3 Fig and Table 2). In addition, the median number of CTCs, as detected by CellSearch, CK^+^/Ki67^+^, CK^+^/Ki67^-^, CK^+^/M30^-^, CK^+^/Vim^+^ and CK^+^/Vim^-^ was significantly increased on PD compared to post-1^st^ cycle values (*p<*0.001, *p<*0.001, *p<*0.001, *p<*0.001, *p<*0.001 and *p<*0.001, respectively; Table 2 and S3 Fig).

### Detection of CTCs and clinical outcome

An objective response (Complete and Partial Response; CR/PR) was achieved in 77 (71.3%) patients while 11 (10.2%) and 14 (13.0%) patients, experienced stable and progressive disease, respectively (Table 1 and S1 File). Patients with disease progression had a significantly higher number of CTCs at baseline as assessed by CellSearch compared to patients who experienced a CR/PR or SD (median 123 CTCs/7.5 ml vs 8 CTCs/7.5 ml vs 12 CTCs/7.5 ml, respectively; *p =* 0.036, S3 Table). Nevertheless, clinical response to treatment was not associated with the presence of any different subpopulation of CTCs (S3 Table). The median PFS and OS for all patients was 6.8 months (95% CI: 6.2–7.5) and 10.8 months (95% CI: 8.8–12.8), respectively.

**Fig 1:**
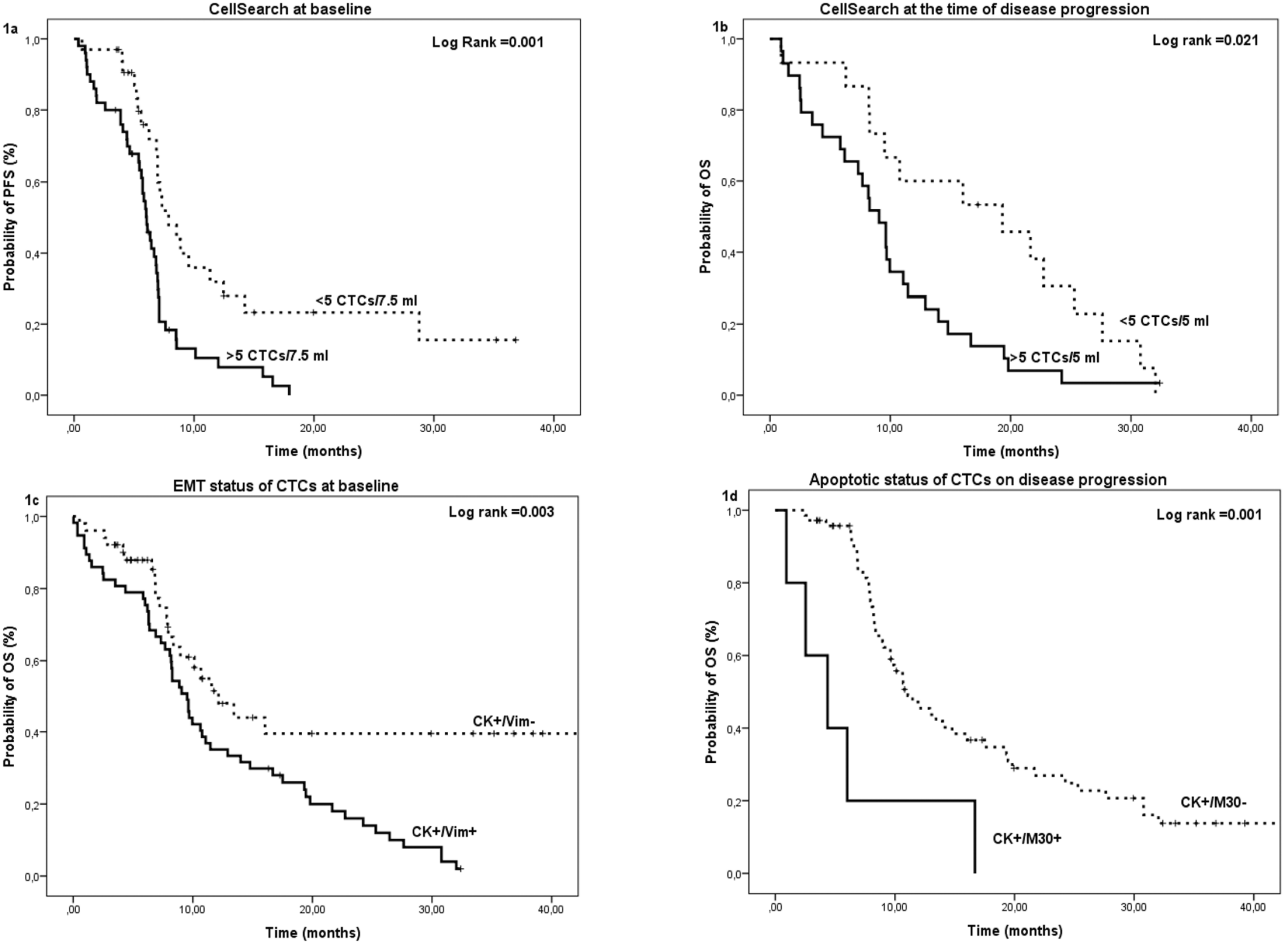
Kaplan Meier curves for PFS and OS. (a) Probability of PFS according to the detection of CTC at baseline by the CellSearch, (b) Probability of OS according to the detection of CTC on disease progression by the CellSearch, (c) Probability of OS according to the detection of EMT phenotype expression at baseline and (e) Probability of OS according to the detection of apoptotic phenotype on disease progression.

Patients with a high number of CTCs had a significantly shorter median PFS compared to patients with a low number of CTCs irrespectively of the time of CTC enumeration [high versus low CTC number at baseline: 6.0 (95% CI: 5.4–6.7) versus 7.9 (95% CI: 5.7–10.1) months (*p =* 0.001) (Fig 1a); high versus low CTC number after one treatment cycle: 5.4 (95% CI: 3.2–7.5) versus 7.0 (95% CI: 6.8–7.2) months (*p =* 0.004)] (S4a Fig). Similarly, patients with a high CTC number had a significantly decreased median OS compared to patients with a low CTC number (high versus low CTC number at baseline: 8.4 (95% CI: 3.2–7.5) versus 21.7 (95% CI: 15.6–27.7) months (*p<*0.001); high versus a low CTC number after one treatment cycle: 8.2 (95% CI: 6.2–10.3) versus 14.8 (95% CI: 10.3–19.3) months (*p =* 0.004) (S4c and S4d Fig) and high versus a low CTC number at the time of PD: 9.1 (95% CI: 7.2–11.0) versus 19.3 (95% CI: 6.6–32.1) moths (*p =* 0.021) (Fig 1b).

Similarly, shorter PFS was observed in patients with CK^+^/Ki67^+^ and CK^+^/Vim^+^ CTCs at baseline (*p*<0.001 and *p* = 0.001; S5a and S5b Fig) and in patients with CK^+^/Ki67^+^, CK^+^/M30^+^ and CK^+^/Vim^+^ CTCs after one treatment cycle (*p* = 0.031, *p*<0.001 and *p* = 0.029; S5c–S5e Fig). Moreover, shorter OS was observed in patients with CK^+^/Ki67^+^, CK^+^/M30^+^ and CK^+^/Vim^+^ CTCs at baseline (*p* = 0.001, *p* = 0.014 and *p* = 0.003; S5f and S5g Fig and Fig 1c) and in patients with CK^+^/M30^+^ CTCs on disease progression (*p* = 0.001; Fig 1d).

### Univariate and multivariate analysis

Univariate analysis revealed that the high number of CTCs as detected by CellSearch or the detection of the different CTC subpopulations (proliferative, apoptotic and/or EMT) as detected by immunofluorescence at baseline and after one treatment cycle or on PD were significantly associated with a shorter PFS and OS (Table 4). In multivariate analysis, adjusting for these factors, only the detection of CTCs at baseline was emerged as an independent factor associated with decreased PFS (HR: 1.9, 95%CI: 0.7–3.6; *p =* 0.032). Moreover, the detection of CTCs on PD, of apoptotic CTCs on PD and of Vim^+^ CTCs at baseline emerged as independent factors associated with decreased OS (HR: 2.1, 95%CI: 1.0–4.5; *p =* 0.043; HR: 6.4, 95% CI: 1.6–25.8; *p =* 0.009; HR: 4.6, 95% CI: 1.2–16.8, *p =* 0.023, respectively) (Table 4).

**Table 4:** Univariate and multivariate Cox regression analysis.

	Univariate Analysis				Multivariate Analysis			
	PFS*		OS**		PFS*		OS**	
	Hazard Ratio (95,0%CI)	*p*-value	Hazard Ratio (95,0%CI)	*p*-value	Hazard Ratio (95,0%CI)	*p*-value	Hazard Ratio (95,0%CI)	*p*-value
**CellSearch at baseline (≥5 vs <5 CTCs)**	2,4 (1,4–4,1)	0,001	3,4 (1,8–6,3)	<0,001	1,9 (0,7–3,6)	0,032	-	-
**CellSearch post 1st cycle (≥5 vs <5 CTCs)**	1,9 (1–3,5)	0,05	-	-	-	-	-	-
**CellSearch on disease progression (≥5 vs <5 CTCs)**	-	-	2,1 (1,1–4,0)	0,033	-	-	2,1 (1,0–4,5)	0,043
**CK**^**+**^**/Ki67**^**+**^ **vs CK**^**+**^**/Ki67**^**-**^ **at baseline**	2,2 (1,4–3,4)	0,001	2,2 (1,4–3,7)	0,002	-	-	-	-
**CK**^**+**^**/Ki67**^**+**^ **vs CK**^**+**^**/Ki67**^**-**^ **post 1st cycle**	1,7 (1,1–2,8)	0,034	-	-	-	-	-	-
**CK**^**+**^**/M30**^**-**^ **vs CK**^**+**^**/M30**^**+**^ **at baseline**	2,4 (1,3–3,2)	0,001	2,7 (1,5–4,8)	0,001	-	-	-	-
**CK**^**+**^**/M30**^**-**^ **vs CK**^**+**^**/M30**^**+**^ **post 1st cycle**	1,9 (1,2–3,3)	0,012	4,3 (1,7–10,9)	0,002	-	-	-	-
**CK**^**+**^**/M30**^**-**^ **vs CK**^**+**^**/ M30**^**+**^ **on disease progression**	-	-	3,0 (1,3–6,9)	0,013	-	-	6,4 (1,6–25,8)	0,009
**CK**^**+**^**/Vim**^**+**^ **vs CK**^**+**^**/Vim**^**-**^ **at baseline**	2,1 (1,3–3,2)	0,001	2,1 (1,3–3,5)	0,003	-	-	4,6 (1,2–16,8)	0,023
**CK**^**+**^**/Vim**^**+**^ **vs CK**^**+**^**/Vim**^**-**^ **post 1st cycle**	1,7 (1,1–2,9)	0,032	-	-	-	-	-	-

Univariate and multivariate progression free survival (PFS) and overall survival (OS) analysis of small cell lung cancer patients by Cox proportional hazards model.

*PFS: Progression-free survival

**OS: Overall survival

## Discussion

The present study, investigated the phenotypic heterogeneity of CTCs in patients with newly diagnosed SCLC as well as their changes during the front-line treatment and on PD. To this end, CTCs were enumerated using the CellSearch platform and their proliferative, apoptotic and/or EMT status was detected by immunofluorescence. The presented data indicate that even one-chemotherapy cycle resulted in a significant decrease of the patients with an increased number of CTCs (≥5 CTCs/7.5 ml) as well as of the absolute number of the different subpopulations of CTCs (CK^+^/Ki67^+^:*p* = 0.001; CK^+^/Ki67^-^:*p<*0.001; CK^+^/M30^+^:*p =* 0.008; CK^+^/M30^-^:*p<0*.008; CK^+^/Vim^+^:*p<*0.001). These observations are in agreement with the widely accepted notion of CTC heterogeneity. It is noteworthy, that there was a clear correlation between the number of patients with CK^+^/Ki67^-^, CK^+^/M30^+^, CK^+^/M30^-^ and CK^+^/Vim^-^ CTCs but not of them with CK^+^/Ki67^+^ and CK^+^/Vim^+^ CTCs and the CTC number detected by CellSearch (Table 3). This observation suggests that the CK^+^/Ki67^+^ and CK^+^/Vim^+^ CTC subpopulations are different from the cells which can be detected by the CellSearch assay and this suggestion is further supported by the finding that CK^+^/Ki67^+^ and CK^+^/Vim^+^ CTCs could be detected in a number of patients with undetectable CTCs by the CellSearch; it is interesting to note that in these particular patients, further immunofluorescence experiments demonstrated the absence of CK^+^/EpCAM^+^ or Vim^+/^EpCAM^+^ explaining, thus, the failure of CellSearch to capture these subpopulations of CTCs. Based on these results, it is obvious that the use of both technologies offers a better detection and characterization of CTCs in patients with SCLC.

Hou *et al*. [16], have already reported that CTCs were present in 85% of the patients with SCLC before chemotherapy and their number was associated with a decreased median PFS and OS [16]. Additionally, PFS and OS were significantly lower in patients with at least one CTM [16]. Similarly, Shi *et al*. using a molecular assay, also demonstrated that the detection of CTCs represents a high risk for both reduced PFS and OS in patients with SCLC [39]. In our study, ≥5 CTCs/7.5 ml were detected in 60.2% of the patients before treatment initiation. Moreover, CTCs detection at baseline was significantly associated with a poor PFS and OS. Our findings, which are in agreement with those of the literature, further support the clinical relevance of CTC enumeration in patients with SCLC, despite the fact that we used a lower cut-off to characterize a blood sample as positive than that used in the Hou’s study [16].

The current study also demonstrated that both the incidence of detection of a high number of CTCs as well as the absolute number of CTCs were significantly reduced after one chemotherapy cycle, reaching the baseline levels on PD. This effect of chemotherapy on CTCs was observed irrespectively of the used detection method. The detection of CTCs after one cycle of chemotherapy seems to indicate that some subpopulations of CTCs are chemo-sensitive whereas others are not. Similar findings have been observed in other tumor types; indeed, we have reported that adjuvant chemotherapy may eliminate CTCs in almost 50% of the patients [40,41]. Moreover, the detection of CTCs after the completion of adjuvant chemotherapy and/or hormone treatment in patients with early breast cancer was associated with a poor patients’ clinical outcome [42,43,44]. Hou *et al*. [16], also showed that after one chemotherapy cycle, PFS and OS were significantly lower in patients with a high number of CTCs (≥50 CTCs/7.5 ml) [16]. Similar results concerning both the PFS and OS were also reported by Naito *et al* [19]. In our study, after one treatment cycle, PFS and OS were significantly lower in patients with detectable CTCs. However, a recent study reported that although CTCs have a useful prognostic role at baseline, only a massive reduction in the CTC number after one chemotherapy cycle, significantly improved prognostic accuracy [45]. In our study, multivariate analysis revealed that the presence of CellSearch-positive CTCs emerged as independent prognostic factor for reduced PFS; moreover, the detection of CTCs with EMT phenotype before treatment initiation and the detection of non-apoptotic CTCs and of CellSearch-positive CTCs at the time of PD emerged as independent prognostic factors associated with reduced OS.

In conclusion, the current study demonstrated an important phenotypic heterogeneity of CTCs in patients with SCLC which is maintained during the treatment with front-line chemotherapy. Moreover, some subpopulations of CTCs seem to be clinically relevant since their detection is associated with patients’ clinical outcome. Moreover, the data strongly support the use of CTCs in patients with SCLC as a dynamic biomarker of treatment efficacy. Subsequent studies have to evaluate whether the phenotypic changes of CTCs during treatment could be associated with a specific molecular and/or genetic profile which could provide information towards a more individualized treatment.

## Supporting information

S1 Fig Flow chart of the study. The flow chart presents the total number of patients included in the study, the number of patients evaluated for their CTCs number according to each method and at each time point.
(TIF)

S2 Fig CK^+^/Ki67^+^ (a) CK^+^/M30^+^ (b) and CK^+^/Vim^+^ (c) CTCs by double immunofluorescent staining. Representative double immunofluorescence images from CTCs expressing CK and a) proliferative (anti-Ki67), b) apoptotic (anti-M30) or epithelial-to-mesenchymal transition (anti-vimentin) markers are shown.
(TIF)

S3 Fig Detection of CTCs in patients with small cell lung cancer patients during front line treatment. The percentage (%) of patients with detectable CTCs during front line treatment and the statistical significance of the change of this positivity are shown in the graph.
(TIF)

S4 Fig Kaplan Meier curves for PFS and OS according to the detection of CTCs by the CellSearch. (a) PFS according to the detection of CTCs by the CellSearch after one treatment cycle; (b) OS according to the detection of CTCs by the CellSearch at baseline and (c) OS according to the detection of CTCs by the CellSearch after one-treatment cycle.
(TIF)

S5 Fig Kaplan Meier curve for PFS and OS according to the detection of different CTCs sub-populations by immunofluorescence. (a) PFS according to the detection of proliferative and (b) epithelial-to-mesenchymal phenotype (EMT) at baseline; (c) PFS according to the detection of proliferative and (d) apoptotic phenotype after one-treatment cycle; and (e) PFS according to the detection of EMT phenotype at the time of disease progression. OS according to the detection of (f) proliferative and (g) apoptotic phenotype at baseline.
(TIF)

S1 Table Detection of different phenotypes of CTCs in patients with <5 CTCs/7,5ml of blood by CellSearch. (PDF)

S2 Table Detection of CTCs subpopulations with immunofluorescence in patients without detectable CTCs by CS (0 CTCs/7,5 ml). (PDF)

S3 Table Objective responses to treatment according to the phenotype of CTCs at baseline. (PDF)

S1 File Patients clinical data and analysis results. (XLSX)
