# Supplementary figures and images for: Phenotypic characterization of circulating tumor cells in the peripheral blood of patients with small cell lung cancer
Source: PLoS One. 2017 Jul 18;12(7):e0181211. doi: 10.1371/journal.pone.0181211 (PMC5515424; doi:10.1371/journal.pone.0181211)

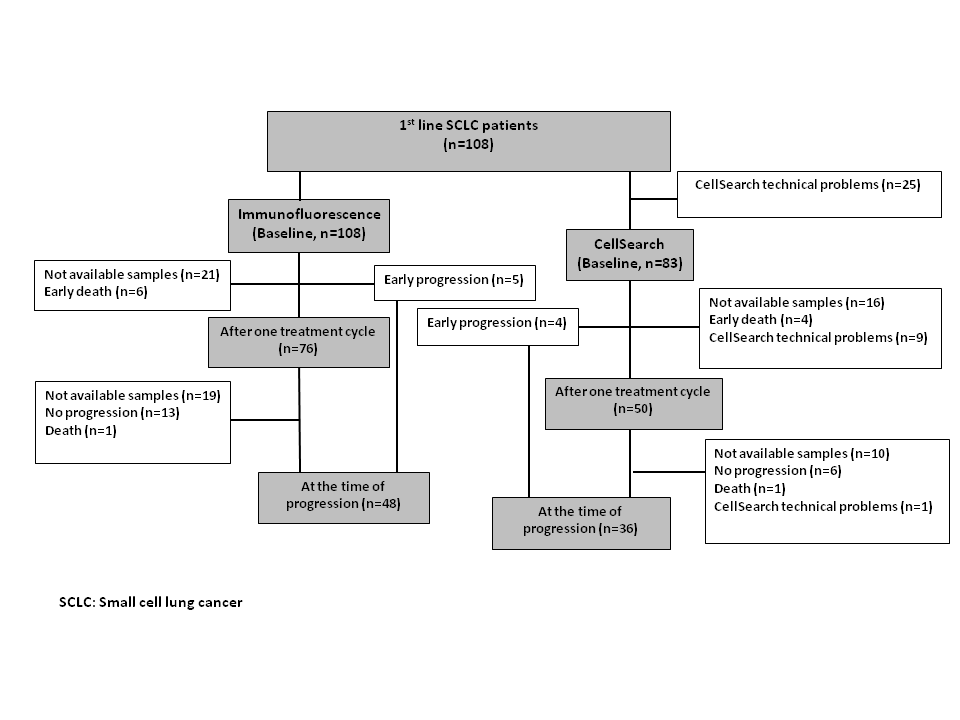

Supplement: S1 Fig — The flow chart presents the total number of patients included in the study, the number of patients evaluated for their CTCs number according to each method and at each time point. (TIF) [file pone.0181211.s001.tif]

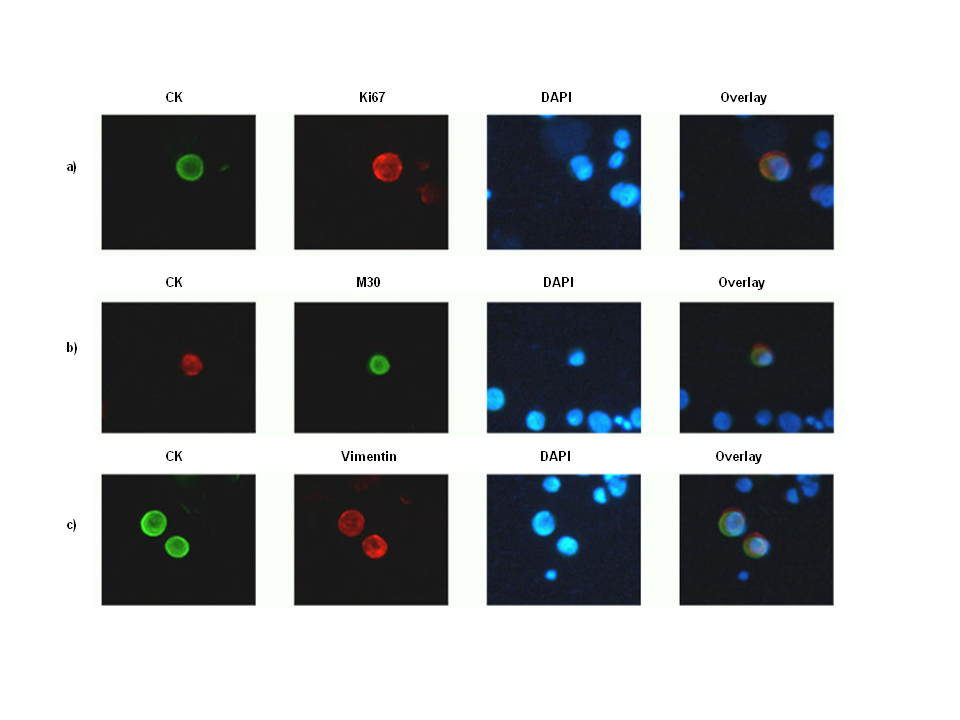

Supplement: S2 Fig — Representative double immunofluorescence images from CTCs expressing CK and a) proliferative (anti-Ki67), b) apoptotic (anti-M30) or epithelial-to-mesenchymal transition (anti-vimentin) markers are shown. (TIF) [file pone.0181211.s002.tif]

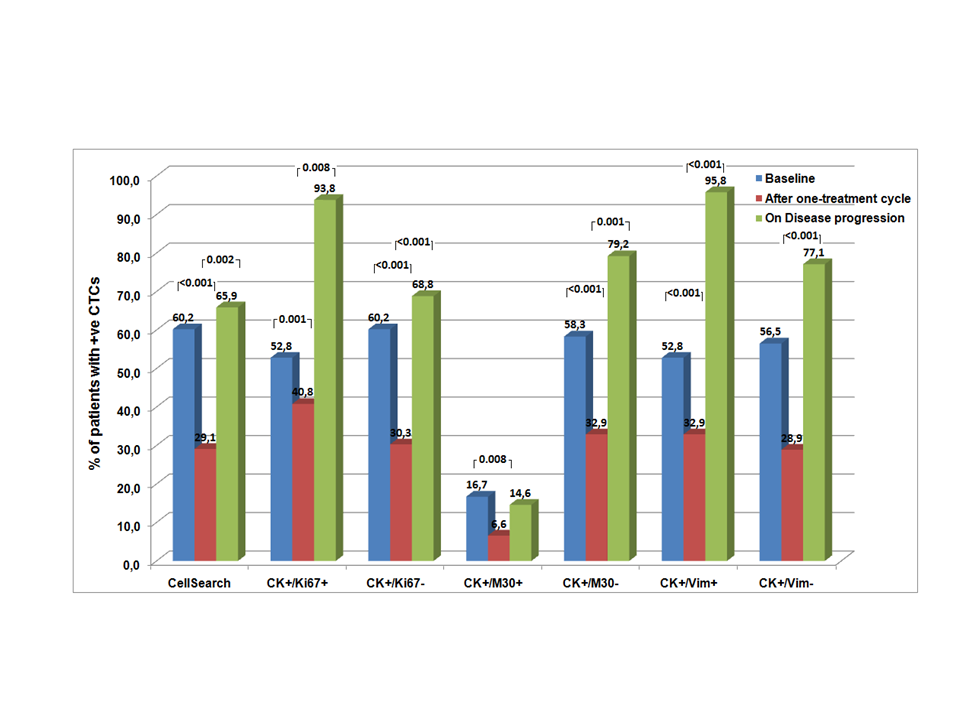

Supplement: S3 Fig — The percentage (%) of patients with detectable CTCs during front line treatment and the statistical significance of the change of this positivity are shown in the graph. (TIF) [file pone.0181211.s003.tif]

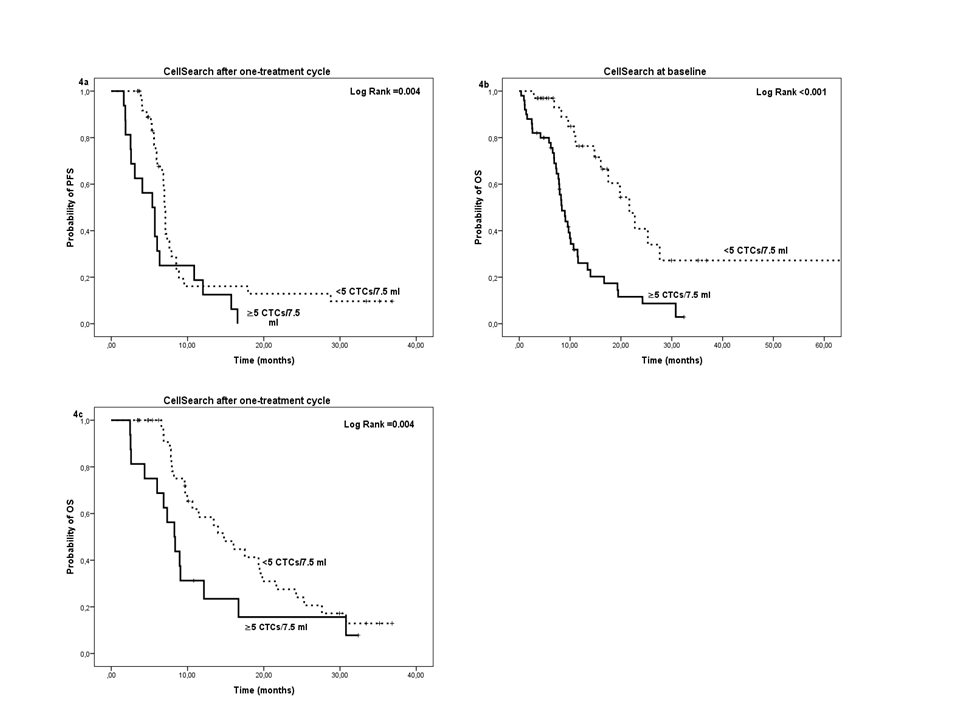

Supplement: S4 Fig — (a) PFS according to the detection of CTCs by the CellSearch after one treatment cycle; (b) OS according to the detection of CTCs by the CellSearch at baseline and (c) OS according to the detection of CTCs by the CellSearch after one-treatment cycle. (TIF) [file pone.0181211.s004.tif]

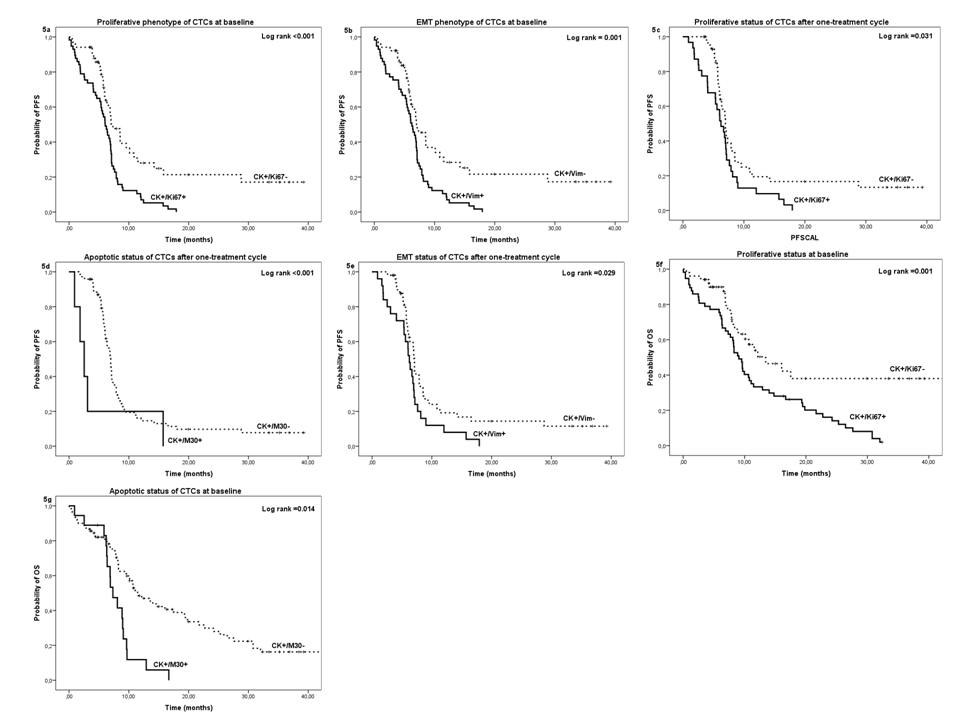

Supplement: S5 Fig — (a) PFS according to the detection of proliferative and (b) epithelial-to-mesenchymal phenotype (EMT) at baseline; (c) PFS according to the detection of proliferative and (d) apoptotic phenotype after one-treatment cycle; and (e) PFS according to the detection of EMT phenotype at the time of disease progression. OS according to the detection of (f) proliferative and (g) apoptotic phenotype at baseline. (TIF) [file pone.0181211.s005.tif]
