# Supplementary material for: Phenotypic characterization of circulating tumor cells in the peripheral blood of patients with small cell lung cancer
Source: PLoS One. 2017 Jul 18;12(7):e0181211. doi: 10.1371/journal.pone.0181211 (PMC5515424; doi:10.1371/journal.pone.0181211)
Supplement: S1 Table — (PDF) [file pone.0181211.s006.pdf]

S1 Table

CTCs /7,5ml (CellSearch)	$CK^{+/}Ki67^{+}$ per 10^{6} PBMCs	$CK^{+}/M30^{+}$ per 10^{6} PBMCs	CK^{+}/Vim^{+} per 10^{6} PBMCs
**<5 (n=33)**	12 (36,4%)	0 (0,0%)	13 (39,4%)
**1-4 (n=11)**	6 (54,5%)	0 (0,0%)	7 (63,6%)
**0 (n=22)**	6 (27,3%)	0 (0,0%)	6 (27,3%)
