# Supplementary material for: Phenotypic characterization of circulating tumor cells in the peripheral blood of patients with small cell lung cancer
Source: PLoS One. 2017 Jul 18;12(7):e0181211. doi: 10.1371/journal.pone.0181211 (PMC5515424; doi:10.1371/journal.pone.0181211)
Supplement: S2 Table — (PDF) [file pone.0181211.s007.pdf]

S2 Table

Patient's No	$CK^+/EpCam^+$	$Vim^+/EpCam^+$	$CK^+/Ki67^+$	$CK^+/Ki67^-$	$CK^+/M30^+$	$CK^+/M30^-$	CK^+/VIM^+	CK^+/VIM^-
**1**	0	0	59	103	0	63	166	77
**2**	0	0	0	0	0	0	0	0
**3**	0	0	0	0	0	0	0	0
**4**	0	0	19	11	0	19	19	12
**5**	0	0	11	16	0	11	10	14
**6**	0	0	17	13	0	19	12	11
**7**	0	0	0	10	0	16	0	11
**8**	0	0	2	0	1	1	4	0
**9**	0	0	0	200	4	186	0	221
**10**	0	0	11	4	2	4	6	1
**11**	0	0	79	64	0	55	0	59
**12**	0	0	0	0	0	0	0	0
**13**	0	0	12	16	0	16	19	11
