# Supplementary material for: Phenotypic characterization of circulating tumor cells in the peripheral blood of patients with small cell lung cancer
Source: PLoS One. 2017 Jul 18;12(7):e0181211. doi: 10.1371/journal.pone.0181211 (PMC5515424; doi:10.1371/journal.pone.0181211)
Supplement: S3 Table — (PDF) [file pone.0181211.s008.pdf]

S3 Table

	CellSearch (N=83)					Immunofluorescence (N=108)								
	**All patients**	**≥5CTC (%) (N= 50)**	**<5CTCs (%) (N= 33)**	**Median (range)**	***p***	**CK$^+$/ Ki67$^+$**	**CK$^+$/ Ki67$^-$**	***p***	**CK$^+$/ M30$^+$**	**CK$^+$/ M30$^-$**	***p***	**CK$^+$/ Vim$^+$**	**CK$^+$/ Vim$^-$**	***p***
**CR/PR**	77 (71,3)	32 (38,6)	28 (33,7%)	8 (0-10000)		39 (36,1)	38 (35,2)		13 (64,0)	64 (59,3)		40 (37,0)	37 (34,3)	
**SD**	11 (10,2)	6 (7,2)	4 (4,8%)	12 (0-4544)	0,003	3 (2,8)	8 (7,4)	0,21	2 (1,9)	9 (8,3)	0,49	3 (2,8)	8 (7,4)	0,084
**PD**	14 (13,0)	9 (10,8)	1 (1,2%)	123 (4-7600)		12 (11,1)	2 (1,9)		3 (2,8)	11 (10,2)		11 (10,2)	3 (2,8)	
**Unknown**	6 (5,6)	3 (3,6)	0 (0,0%)	89 (18-228)		3 (2,8)	3 (2,8)		0 (0,0)	6 (5,6)		3 (2,8)	3 (2,8)	
